# Membranoproliferative glomerulonephritis associated with Rosai-Dorfman disease 

**DOI:** 10.5414/CNCS108856

**Published:** 2017-08-30

**Authors:** Keisuke Sugimoto, Satoshi Ueda, Mitsuru Okada, Tsukasa Takemura

**Affiliations:** Department of Pediatrics, Kinki University Faculty of Medicine, Osaka, Japan

**Keywords:** membranoproliferative glomerulonephritis, Rosai-Dorfman disease, mizoribine, cytokines, Langerhans-cell histiocytosis, children

## Abstract

Rosai-Dorfman disease is also known as sinus histiocytosis with massive lymphadenopathy. Extranodal Rosai-Dorfman disease has been reported in ~ 43% of cases; the most frequent extranodal sites – skin, soft tissue, bone, respiratory tract, and eye – are usually involved in association with lymphadenopathy. Lack of lymph node involvement is rare, especially when patients manifest renal disease. Here, we describe a patient who developed membranoproliferative glomerulonephritis when lymphadenopathy was absent. During follow-up for sinus histiocytosis, a 7-year-old Japanese boy developed proteinuria and hematuria. No renal abnormality was present in ultrasound imaging. Histologic examination of a renal biopsy specimen disclosed moderate mesangial proliferation, focal thickening of glomerular capillary walls, and mesangial interposition. Mononuclear cells infiltrated the interstitium. Immunofluorescence showed intense IgG, C3, and C4 reactivity in portions of the mesangium and glomerular capillary walls. Electron microscopy depicted nodular deposits in mesangial, endocapillary, and subepithelial areas. Immunohistochemistry for S-100 protein, CD68, and lysozyme was positive within the interstitium. CD1a staining was absent. These findings were diagnostic for membranoproliferative glomerulonephritis. Multidrug therapy, including methylprednisolone and mizoribine, improved urinary findings and induced complete remission of both diseases. To the best of our knowledge, this is the first report of Rosai-Dorfman disease complicated by renal disease in the absence of concurrent nodal involvement. Clinicians should be alert to this diagnostic possibility.

## Introduction 

Rosai-Dorfman disease (RDD), also known as sinus histiocytosis with massive lymphadenopathy, was recognized first as a distinct clinicopathologic entity by Rosai and Dorfman in 1969 [1]. Although the causes of RDD are not fully understood, cytokine-mediated migration of monocytes may be responsible for histiocytic accumulation and activation. The proliferation of histiocyte characteristics of RDD, a rare non-neoplastic disease, occurs mostly in lymph nodes. The disease most frequently affects children and young adults [2]. In classic cases, patients with RDD often present with cervical lymphadenopathy, fever, malaise, weight loss, mild anemia, leukocytosis, an elevated sedimentation rate, and polyclonal hypergammaglobulinemia. Extranodal RDD has been noted in ~ 43% of cases; the most frequent sites are skin, soft tissue, multiple foci in bone, upper respiratory tract, eye, and retro-orbital tissues. Extranodal manifestation of RDD without involvement of lymph nodes is rare, especially in patients with renal disease. Two forms of renal disease have been reported in this context [3]; among 423 RDD cases in a registry, 4 were associated with glomerulonephritis and 10 presented as infiltrative renal masses [3, 4]. 

Membranoproliferative glomerulonephritis (MPGN), a form of chronic glomerulonephritis occurring in children and adults, can be either idiopathic or secondary. Cases of secondary MPGN include infections, autoimmune disease, chronic liver disease, malignant neoplasia, lymphoproliferative disorders causing monoclonal gammopathy, and essential cryoglobulinemia. MPGN, therefore, is considered glomerular injury seen in a variety of disease processes that share a common pathogenetic mechanism, rather than a single disease entity [5]. 

Langerhans cell histiocytosis (LCH) is a rare disorder categorized as a class I histiocytosis syndrome. Immunohistochemically, the Langerhans cells in LCH frequently express CD68; in addition, immunostaining for both S-100 neuroprotein and CD1a is required for diagnosis of LCH [6]. In contrast, CD1a expression is absent in RDD. Both of these histiocytoses show a strong macrophage antigen expression. As for renal complications in LCH, membranous nephropathy and MPGN have been reported being attributed to abnormalities of the immune system [7, 8]. Just as certain nephropathies may occur together with lymphadenopathy, such as that in RDD and LCH, development of MPGN in our present patient would likely be related to RDD – even though lymphadenopathy has resolved. To our knowledge, however, this is the first report of such a case. 

## Case presentation 

**Figure 1.**
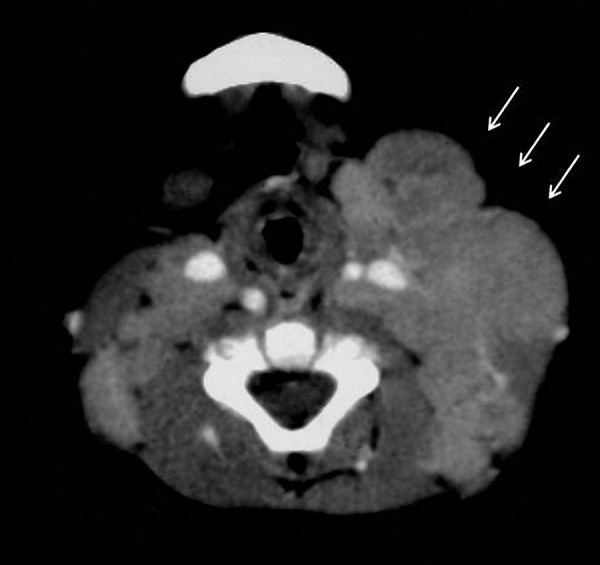
Radiologic findings in the patient at the age of 9 months. Axial contrast-enhanced computed tomography shows left cervical adenopathy (arrows).

**Figure 2.**
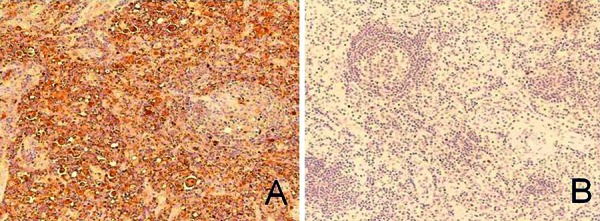
Immunohistochemistry findings. A cervical lymph node biopsy specimen displays nodal involvement by RDD. Immunohistochemical staining for S-100(+) is positive (A), while staining for CD1a is negative (original magnification, ×400) (B).

A 7-year-old Japanese boy was hospitalized upon recurrence of proteinuria and hematuria. When he was 9 months old, he developed cervical lymph node enlargement and persistent fever. Histopathologically, a cervical lymph node showed changes diagnostic for RDD (Figure 1 and Figure 2). Although lymphadenopathy resolved with a course of oral prednisolone (PSL), several recurrences required additional courses. One of these, at 4 years of age, was accompanied by mild proteinuria and hematuria; PSL therapy abolished lymphadenopathy and urinary abnormalities. After PSL was discontinued at the age of 5 years, renal disease became evident at 7 years. 

No family history of autoimmune renal disease could be obtained. No cervical lymph nodes or other peripheral nodes were enlarged. Oliguria, ascites, and edema were absent. Physical finding upon admission included height, 115 cm (–1.3 SD); weight, 22 kg (–0.5 SD); blood pressure, 110/70 mmHg; pulse, 80/min; temperature, 37 °C; respiratory rate, 20/min. 

Creatinine clearance was normal (120.5 mL/min/1.73 m^2^) with no abnormal findings. Peripheral white cell count including the rate of eosinophil cells was normal. Most laboratory blood examinations, include electrolytes, serum creatinine, and blood urea nitrogen. Aspartate aminotransferase was 18 IU/L; (normal range, 14 – 20), and alanine aminotransferase activities was 23 IU/L; (normal range, 10 – 40). Lactate dehydrogenase activity and creatinine kinase activity were normal. Serologic analysis for antinuclear antibody and MPO-ANCA were negative. Serum complement component C3 was 101 mg/dL; (normal range, 82 – 145); C4, 18 mg/dL (normal range, 12 – 33); and total complement activity (CH50) was 45.4 U/mL (normal range, 24.2 – 52.8). Circulating immune complexes (CIC) were 1.5 mg/mL (normal range, below 3.0). Serum IgG, IgA, IgM, and IgE concentrations were normal. An infectious work-up came back negative for hepatitis B and C. Serum interleukin-1 beta (IL-1β) was elevated (89 pg/mL; normal range, < 10), as was soluble interleukin-2 receptor (sIL-2R; 1,705 U/mL; normal range, 122 – 496). Serum interleukin-2 (IL-2) was < 8 U/mL (normal range; < 8); interleukin-4 (IL-4), 5.7 U/mL (normal range; < 6) and interleukin-6 (IL-6), 1.3 U/mL (normal range; < 4). Tumor necrosis factor-alpha (TNF-α) was 2.1 pg/mL (normal range; < 2.8). 

**Figure 3.**
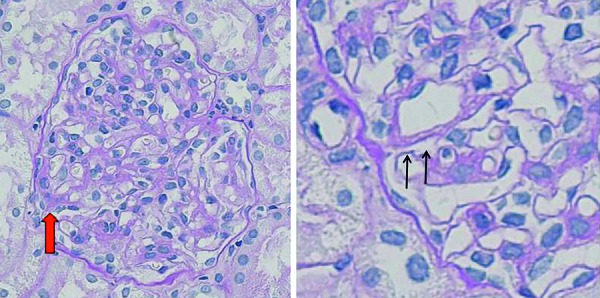
Histologic findings at 7 years. The renal biopsy specimen shows enlargement of a glomerulus with mesangial proliferation, thickening of segmental capillary walls, mesangial interposition (thick red arrows) and double contours (thin arrow). Periodic acid-Schiff (PAS) stain (original magnification, ×400).

**Figure 4.**
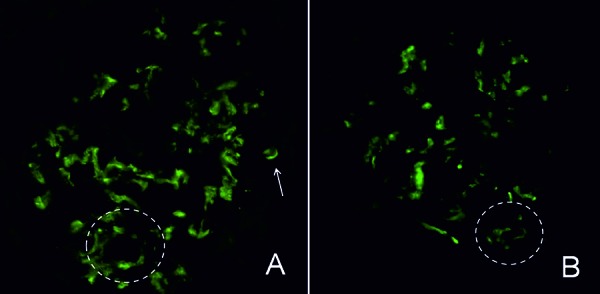
Immunofluorescence findings. Immunofluorescence shows intense IgG (A) and C3 (B) deposition in the mesangium and the focally in glomerular capillary walls (circle and arrow) (original magnification, ×400). No glomerular IgA, IgM, C1q, or fibrinogen deposition is demonstrated.

**Figure 5.**
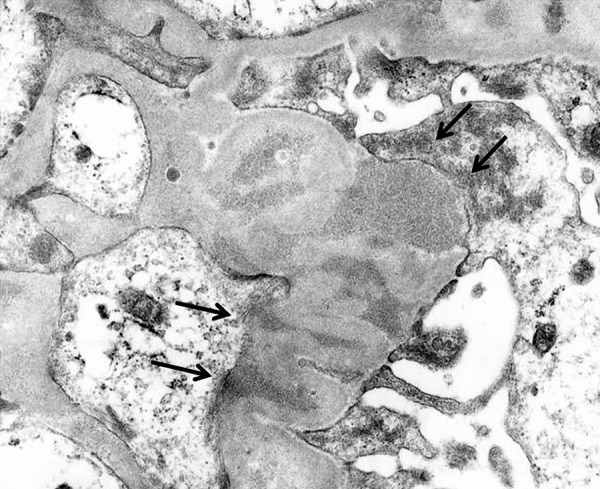
Electron microscopic findings at 7 years. Nodular deposits (arrows) are shown in mesangial, endocapillary, and subepithelial areas (original magnification, ×7,000).

**Figure 6.**
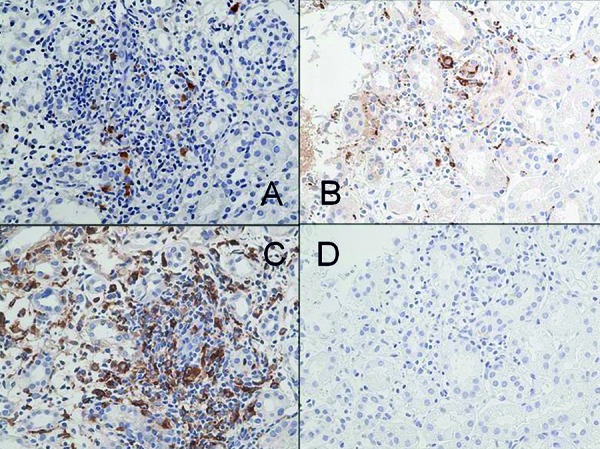
Immunohistochemical findings in the kidney upon repeat biopsy. Infiltration of mononuclear cells in the interstitium identified by staining for S-100 (A), CD68 (B), and lysozyme (C). CD1a staining is negative in the same area (magnification, ×400) (D).

Mild to moderate proteinuria (0.5 g/day) was noted as was hematuria. Ultrasonographically renal size and shape were normal, and mass lesions were absent. Histologic examination of a renal biopsy specimen disclosed moderate mesangial proliferation, focal thickening of glomerular capillary walls, and mesangial interposition (Figure 3). Mononuclear cells infiltrated the interstitium; plasma cell infiltration was absent in both glomerular and interstitial areas. Interstitial fibrosis, atrophy or other injury of tubular cells, and necrosis all were absent. Immunofluorescence showed intense IgG, C3, and C4 reactivity in portions of the mesangium and glomerular capillary walls (Figure 4) but no reactivity for IgA and IgM or other complement components such as C1q. Electron microscopy disclosed nodular deposits in mesangial, endocapillary, and subepithelial areas (Figure 5). Immunoreactivity for S-100 protein, CD68, and lysozyme was present within the interstitium. CD30 and CD1a reactivity was absent (Figure 6). Taken together, these findings confirmed a diagnosis of membranoproliferative glomerulonephritis. 

Treatment was initiated with methylprednisolone pulse therapy (30 mg/kg/day, 3 day/week × 2 weeks), followed by oral PSL (2 mg/kg/day); mizoribine (7 mg/kg/day); an angiotensin-converting enzyme inhibitor; and warfarin. Urinary findings gradually improved, with remission 4 months after treatment was initiated. After tapering of PSL, low-dose PSL and other drugs were continued. A second renal biopsy was performed after 1 year. Histologic examination disclosed mild mesangial proliferation, without focal thickening of glomerular capillary walls. Mononuclear cell infiltration was absent, although slight focal fibrosis was present within the interstitium. PSL and mizoribine were discontinued 2 years after initiation of therapy; only the ACE inhibitor was continued on the basis of an antiproteinuric effect and reduction of glomerular pressure. The patient remains in complete remission with no lymph node enlargement. 

## Discussion 

Sinus histiocytosis with massive lymphadenopathy is a diffuse disorder that can involve numerous organs including eye, bone, central nervous system, ear, throat, upper respiratory tract, liver, skin, salivary gland, and testis. Renal disorders complicating RDD are very uncommon [9, 10, 11]. Renal failure in RDD patients induced by infiltrating renal masses has been reported [12, 13]. MPGN is characterized by production of a variety of cytokines, glomerular mesangial cell alterations, extracellular matrix accumulation, and thickening of glomerular capillary walls with mesangial interposition. In a model of MPGN using rats with Thy-1 nephritis, serum IL-1β and IL-6 concentrations were increased [14]. Sakallioglu et al. [15] reported a boy with RDD complicated by nephrotic syndrome, which can be associated with excesses of cytokines such as TNF-α, IL-1β, and IL-6 as well as polyclonal gammaglobulinemia including IgE [16, 17, 18]. Immunemechanisms associated with cytokines could have triggered nephrotic syndrome in Sakallioglu’s case [15]. Our patient also showed elevated IL-1β, suggesting cytokine-induced glomerular cell injury/activation as a possible cause of glomerular pathologic change. Another case with renal involvement involved a patient with LCH; renal insufficiency was accompanied by renal infiltrates made up of CD1a-positive cells, which are characteristic of LCH [19]. In our patient’s renal specimen, S-100 protein, CD68, and lysozyme-stained cells were present within the interstitium. A relationship between the cells infiltrating the renal interstitium and the glomerular changes could be suspected. 

Since RDD is a self-limited disease with a very good outcome in most patients, use of immunosuppressive therapy should be restricted to patients with life-threatening disease, who are un-responsiveness to steroids, or who have had multiple relapses [20]. Systemic corticosteroids are usually helpful in decreasing nodal enlargement and symptoms. In our patient, low-dose steroid therapy was essential for suppressing recurrences of RDD before the patient received multiple-drug therapy for MPGN. Such therapy, which included mizoribine, improved urinary findings and induced complete remission of both RDD and MPGN. Although use of mizoribine for RDD has not yet been reported, this immunosuppressant drug shows promise in maintenance of remission with few side effects, especially in children. 

## Conclusion 

In summary, renal dysfunction is rare in RDD. This is the first report showing that treatment with prednisolone and mizoribine could achieve remission in a patient with both RDD and MPGN. 

## Consent 

Written informed consent was obtained from the patient for publication of this case study and any accompanying images. 

## Conflict of interest 

The authors declare no conflict of interest. 

## Acknowledgments 

This study received no grant support.
